# Thiolated Mesoporous Silica Nanoparticles as an Immunoadjuvant to Enhance Efficacy of Intravesical Chemotherapy for Bladder Cancer

**DOI:** 10.1002/advs.202204643

**Published:** 2023-01-13

**Authors:** Cheng‐Che Chen, Yu‐Chen Fa, Yen‐Yu Kuo, Yi‐Chun Liu, Chih‐Yu Lin, Xin‐Hui Wang, Yu‐Huan Lu, Yu‐Han Chiang, Chia‐Min Yang, Li‐Chen Wu, Ja‐an Annie Ho

**Affiliations:** ^1^ BioAnalytical Chemistry and Nanobiomedicine Laboratory Department of Biochemical Science and Technology National Taiwan University 10617 Taipei Taiwan; ^2^ Department of Urology Taichung Veterans General Hospital 40705 Taichung Taiwan; ^3^ Department of Chemistry National Tsing Hua University 300044 Hsinchu Taiwan; ^4^ Instrumentation Center National Taiwan University 10617 Taipei Taiwan; ^5^ Department of Chemistry National Taiwan University 10617 Taipei Taiwan; ^6^ Frontier Research Center on Fundamental and Applied Sciences of Matters National Tsing Hua University 300044 Hsinchu Taiwan; ^7^ Department of Applied Chemistry National Chi Nan University Puli Nantou 54561 Taiwan; ^8^ Center for Emerging Materials and Advance Devices National Taiwan University 10617 Taipei Taiwan; ^9^ Center for Biotechnology National Taiwan University 10617 Taipei Taiwan

**Keywords:** bladder cancer, intravesical therapy, mesoporous silica nanoparticle, mitomycin C, thiol modification

## Abstract

The characteristics of global prevalence and high recurrence of bladder cancer has led numerous efforts to develop new treatments. The spontaneous voiding and degradation of the chemodrug hamper the efficacy and effectiveness of intravesical chemotherapy following tumor resection. Herein, the externally thiolated hollow mesoporous silica nanoparticles (MSN‐SH(E)) is fabricated to serve as a platform for improved bladder intravesical therapy. Enhanced mucoadhesive effect of the thiolated nanovector is confirmed with porcine bladder. The permeation‐enhancing effect is also verified, and a fragmented distribution pattern of a tight junction protein, claudin‐4, indicates the opening of tight junction. Moreover, MSN‐SH(E)‐associated reprogramming of M2 macrophages to M1‐like phenotype is observed in vitro. The antitumor activity of the mitomycin C (MMC)‐loaded nanovector (MMC@MSN‐SH(E)) is more effective than that of MMC alone in both in vitro and in vivo. In addition, IHC staining is used to analyze IFN‐*γ*, TGF‐*β*1, and TNF‐*α*. These observations substantiated the significance of MMC@MSN‐SH(E) in promoting anticancer activity, holding the great potential for being used in intravesical therapy for non‐muscle invasive bladder cancer (NMIBC) due to its mucoadhesivity, enhanced permeation, immunomodulation, and prolonged and very efficient drug exposure.

## Graphical abstract

The nanovectors, mitomycin C (MMC)‐loaded, externally thiolated hollow mesoporous silica nanoparticles (MSN‐SH(E)), are confirmed to be helpful in promoting anticancer activity, holding the great potential for being used in intravesical therapy for non‐muscle invasive bladder cancer due to its mucoadhesivity, enhanced permeation, immunomodulation and prolonged and very efficient drug exposure.

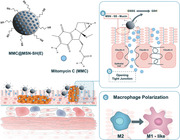

## 1 Introduction

Bladder cancer is one of the most common cancers worldwide with a high incidence and mortality rate,^[^ ^1^ ^]^ being ranked the fourth newly diagnosed cancer and the eighth most common cancer cause in 2020 in the United States.^[^ ^1^ ^]^ Bladder cancer is among the most costly of all cancers to treat.^[^ ^2^ ^]^ About 75% of bladder cancer patients suffered from non‐muscle invasive bladder cancer (NMIBC),^[^ ^3^ ^]^ a challenging disease characterized by a tendency for recurrence and capacity for progression. Nearly 50% to 90% patients diagnosed with NMIBC will recur and progress within five years of their initial diagnosis despite transurethral resection of bladder tumor (TURBT).^[^ ^4^, ^5^ ^]^ Intravesical chemotherapy and Bacillus Calmette–Guerin (BCG) immunotherapy are the most commonly used treatments and/or prophylaxis for NMIBC^[^ ^6^, ^7^ ^]^ that help to prevent recurrence after receiving transurethral resection of bladder tumor (TURBT).^[^ ^8^ ^]^ Though MMC revealed a 10–50% reduction of recurrence,^[^ ^6^, ^9^, ^10^ ^]^ the instilled MMC was found to be degraded nonenzymatically by the acidity of urine (pH < 6),^[^ ^11^ ^]^ and/or enzymatically by the bladder endogenous quinone reductase.^[^ ^12^ ^]^ Bladder exposure to MMC during medical treatment may also be associated with factors including residual urine volume, urine production rate, and removal by systemic absorption and degradation.^[^ ^9^ ^]^ It has also been shown that the dosage and concentration of MMC in urine affected the uptake of MMC by the bladder tissue and the efficacy during intravesical therapy for superficial bladder cancer.^[^ ^13^ ^]^

Attempts have been made to boost the efficacy of mitomycin C (MMC) by, for example, introducing a short‐term intensive schedule of MMC administration to increase MMC solubility at elevated temperature,^[^ ^14^, ^15^ ^]^ or the application of MMC‐hydrogel.^[^ ^16^ ^]^ In recent years, mucoadhesive particles (MAPs) and mucopenetrating particles (MPPs) were utilized to deliver chemodrugs, providing prolonged drug residence time at mucosa or enhancing mucosal permeation to epithelium,^[^ ^17^ ^]^ respectively. Mucosal membranes of the bladder, for example, are the destination of mucoadhesive particles (MAPs), while mucopenetrating particles (MPPs) are able to accomplish deeper penetration of the mucus gel layer. Combination of mucoadhesive and mucopenetrating properties on one nanovector is anticipated to be advantageous. It was previously reported that the mucoadhesive effect that mediated by the formation of disulfide bonds with cysteine‐rich mucin glycoproteins^[^ ^18^, ^19^ ^]^ assisted non‐invasive molecular absorption and enhanced permeation.^[^ ^19^, ^20^ ^]^ Furthermore, auxiliary agents holding properties of mucoadhesion, and mucopenetration, such as thiolated polymers (thiomers),^[^ ^19^ ^]^ chitosan^[^ ^21^ ^]^ and thiolated cyclodextrin,^[^ ^22^ ^]^ have been addressed to improve the delivery of drugs that often suffer from low drug solubility and drug intestinal absorption.^[^ ^22^, ^23^ ^]^

On the other hand, numerous delivery and targeting strategies using functionalized mesoporous silica nanoparticles (MSNs) have been deployed on cancer treatments.^[^ ^24^, ^25^, ^26^ ^]^ MSNs featuring high surface area, large pore volume and versatile chemistry of surface modification are promising for delivering drugs,^[^ ^26^, ^27^, ^28^ ^]^ and the MSNs with hollow morphology render extended loading capacity and appear to be promising drug vectors. A previous report shows that (functionalized) MSNs may interact with immunocompetent cells^[^ ^27^ ^]^ and elicit the expression of marker cytokines of anti‐tumor M1 macrophage.^[^ ^28^ ^]^ It is intriguing to examine the immunomodulatory effect of thiolated MSNs on regulating tumor immune microenvironment (TIME). In addition, an understanding of the thiolated MSNs‐mediated macrophage polarization may provide insights on the regulation of tumor immune microenvironment (TIME), benefiting the development of MSN‐based immunotherapy.

**Figure 1:**
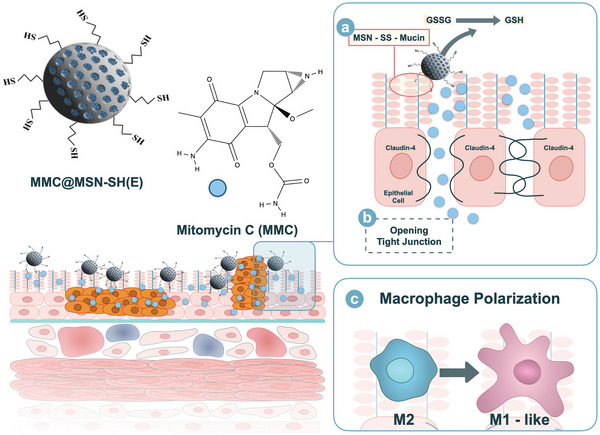
Schematic diagram of MMC@MSN‐SH(E) nanoformulation for the treatment of non‐muscle invasive bladder cancer. Possible therapeutic mechanisms include a) mucoadhesion, b) permeation enhancement, and c) macrophage polarization.

Taken together, we were motivated to develop a new medical strategy that combines the intravesical chemotherapy (with MMC) with immunotherapy (via macrophage polarization) and thiolated MSN (as nanovector) to enhance the treatment efficiency by deploying thiol‐modified, MMC‐loaded MSNs. Mesoporous MMT‐2 silica,^[^ ^29^, ^30^, ^31^ ^]^ a type of MSNs with hollow morphology and a thin mesoporous silica shell, was chosen and synthesized for this study. The external surface of the MSNs was grafted with mercaptopropylsilyl (MPS) groups and resulted in the externally thiolated MSNs, designated as externally thiolated hollow mesoporous silica nanoparticles (MSN‐SH(E)). The interior mesopore surface of MSN‐SH(E) was optionally functionalized with fluorescein isothiocyanate (FITC) to facilitate the exploration of possible targeting effects through enhanced mucoadhesion^[^ ^25^, ^26^ ^]^ by fluorescent imaging. The sample of MSN‐SH(E) was then loaded with MMC to treat NMIBC. The enhanced mucoadhesive and mucopenetrating properties of the MMC‐loaded nanovector were evaluated and the mechanism underlined was also investigated. Subsequently, the sample of MSN‐SH(E) was loaded with MMC to treat NMIBC, and the anticancer activity was examined both in vitro and in vivo. Moreover, by analyzing polarization‐associated biomarkers, MSN‐SH(E) were found to reprogram pro‐tumor M2‐like macrophages into M1‐like phenotypes. The results demonstrated a successful combined chemo/immunotherapy by utilizing MMC‐loaded thiolated MSNs with improved efficacy, owing to better mucoadhesivity, enhanced permeation, prolonged and very efficient drug exposure, as a first strike against NMIBC. The schematic diagram of MMC@MSN‐SH(E) nanoformulation for treating non‐muscle invasive bladder tumors is illustrated in **Figure** **1**.

## 2 Results and Discussion

### 2.1 Characterization of MSNs Materials

**Figure 2:**
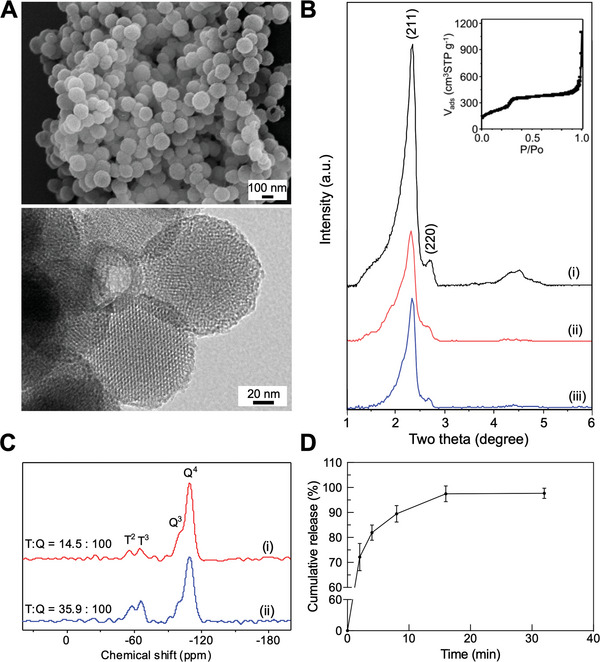
Characterization of MSNs materials. A) SEM and TEM images of B‐MSN, B) XRD pattern and nitrogen physisorption isotherm (inset) of B‐MSN (i), MSN‐SH(E) (ii) and MSN‐SH(E)/FITC(I) (iii), C) ^29^Si MAS NMR spectra of MSN‐SH(E) (i) and MSN‐SH(E)/FITC(I) (ii), and D) releasing profiles. All the results are shown as mean ± SD, *n* = 3.

The mesoporous MMT‐2 silica used herein is a type of MSNs with hollow morphology and thin mesoporous silica shell.^[^ ^29^, ^30^, ^31^ ^]^ The scanning electron microscope (SEM) and transmission electron microscope (TEM) images of B‐MSN (**Figure** **2**A) confirmed the size of spherical nanoparticles around 90–120 nm and the thin mesostructured silica shell. The sample exhibited sharp XRD characteristic reflections of the cubic *Ia3d* structure with unit cell parameter of 9.5 nm and a type IV nitrogen physisorption isotherm with a steep step at relative pressure of 0.30, referring to a mesopore diameter of 2.9 nm (Figure 2B(i)). The specific surface area and mesopore volume of B‐MSN were 950 m^2^ g^−1^ and 0.71 cm^3^ g^−1^, respectively, obtained by analyzing the isotherm using the Brunauer–Emmett–Teller (BET) method ^[^ ^32^ ^]^ and from the amount of nitrogen molecules adsorbed at a relative pressure of 0.9 at 77 K. The ordered mesostructure retained after functionalization of the external surface with MPS groups (i.e., MSN‐SH(E)) (Figure 2B(ii)) and additionally the mesopore surface with covalently linked FITC (i.e., MSN‐SH(E)/FITC(I)) (Figure 2B(iii)). The solid‐state ^29^Si MAS NMR spectra of MSN‐SH(E) and MSN‐SH(E)/FITC(I) exhibited two types of peaks that were associated to Q*^n^* groups (Si(OSi)*_n_* (OH)_4−_ *_n_*)) from the B‐MSN and T*^n^* groups (C‐Si(OSi)*_n_* (OH)_3−_ *_n_*)) attributed to the silicon atoms of the MPS and FITC‐conjugated groups (Figure 2C). The integration ratios of the T and Q peaks for MSN‐SH(E) and MSN‐SH(E)/FITC(I) were 14.5:100 and 35.9:100, respectively. The quantitative analysis was in line with the TGA results (data not shown). We also determined the p*Ka*, stability of the MPS groups and quantification of the thiol groups on MSN‐SH(E), details are provided in Figure S1, Supporting Information.

In this study, MSN‐SH(E) was used to encapsulate MMC to treat NMIBC, for which MMC has to be released from the nanovector during the treatment. To confirm it, MMC@MSN‐SH(E) was dispersed in PBS and the absorbance of supernatant at 364 nm at designated times was recorded. The release profile (Figure 2D) suggested that around 98% of the encapsulated MMC were released within 16 min.

### 2.2 Mucoadhesive Effect of MSN‐SH(E)

**Figure 3:**
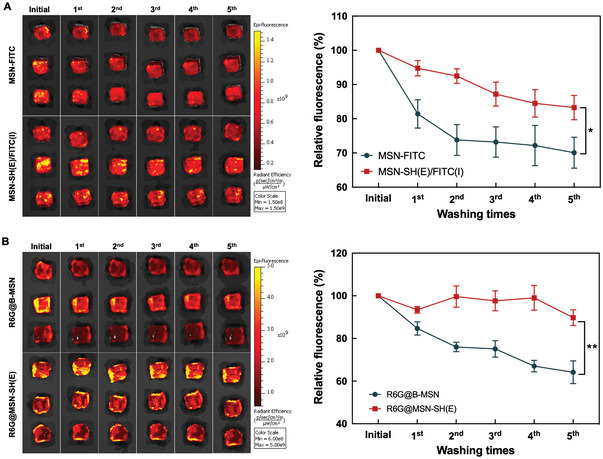
Mucoadhesive effect of various MSNs. IVIS images of porcine bladder wall incubated in PBS (pH 7.4) containing A) MSN‐SH(E)/FITC(I) and MSN‐FITC B) R6G@B‐MSN and R6G@MSN‐SH(E). Five times washing with PBS proceeded after 2 h incubation of various MSNs. All the results are shown as mean ± SD, *n* = 3 (*p < 0.05 and **p < 0.001).

The mucoadhesive effect of thiolated MSNs were examined by dispensing the FITC‐modified MSNs with or without surface thiol groups (i.e., MSN‐SH(E)/FITC(I) and MSN‐FITC) on porcine bladder. As shown in **Figure** **3**A, the sample treated with MSN‐SH(E)/FITC(I) showed stronger residual fluorescence as compared to the one with MSN‐FITC (83.26 ± 3.54% versus 70.07 ± 4.52%), suggesting that MSN‐SH(E)/FITC(I) was more resistant against washing off, most likely due to its firm attachment to the inner lining of the bladder. To further confirm the mucoadhesive ability of MSN‐SH(E), the two FITC‐free MSNs (i.e., B‐MSN and MSN‐SH(E)) were non‐covalently loaded with Rhodamine 6G (R6G) as a fluorescent model drug, and the release of R6G as a function of time was measured. As shown in Figure 3B, a prolonged R6G release for MSN‐SH(E) was observed (89.74 ± 3.69% for R6G@MSN‐SH(E) versus 64.16 ± 5.32% for R6G@B‐MSN of relative fluorescence intensity after repetitive washing), again confirming the mucoadhesive ability of MSN‐SH(E).

**Figure 4:**
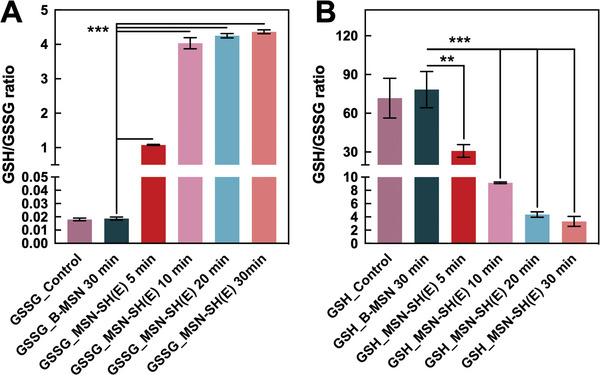
Thiol/disulfide exchange of MSN‐SH(E) with GSSG and GSH. MSN‐SH(E) (2 mg mL^−1^) was reacted with A) GSSG (45 µm) or B) GSH (45 µm) for various periods of time, and the GSH/GSSG ratios of the solutions obtained after centrifugation were determined by fluorescence method. B‐MSN was used as a reference sample. All the results are shown as mean ± SD, *n* = 3 (**p < 0.01 and ***p < 0.001).

Moreover, as aforementioned, one of the mechanisms for mucoadhesion is through disulfide bond formation between the thiol groups of thiomers and mucosa by thiol/disulfide exchange^[^ ^33^ ^]^ and an oxidative process.^[^ ^34^ ^]^ It has been reported that the nucleophilic attack of ionized form of the polymer‐attached cysteine thiol groups on GSSG takes place quickly,^[^ ^20^ ^]^ enabling the formation of disulfide bridges between the thiomers and cysteine‐rich mucin glycoproteins.^[^ ^18^, ^19^ ^]^ Beside, Andreas Bernkop‐Schnürch's group (1999)^[^ ^35^ ^]^ reported that an oxidation process with hydrogen peroxide triggered the formation of disulfide linkages between the thiol groups of thiomer and cysteine moiety of mucin. In this study, the capability of MSN‐SH(E) to form disulfide bonds with mucosa through thiol/disulfide exchange was examined by allowing MSN‐SH(E) to react either GSSG or GSH. The concentration of thiol‐related groups in the reaction mixtures was determined at different periods of reaction time using a fluorescence method. As shown in **Figure** **4**A, MSN‐SH(E) reacted with GSSG and started to generate GSH (with GSH/GSSG ratio of 1.07) after a reaction time of 5 min. A maximum amount of GSH was observed at about 10 min. For comparison, B‐MSN was not capable of converting GSSG to GSH. Obviously, the reaction between the thiol groups on MSN‐SH(E) and GSSG led to a significant degree of thiol/disulfide exchange, rendering MSN‐SH(E) the capability of interacting with mucin 1‐overexpressed urothelial cancer cells,^[^ ^36^ ^]^ resulting in mucin‐targeted therapy. This was in accordance with a previous study showing that thiomers formed disulfide bonds with cysteine‐rich region mucosa.^[^ ^18^ ^]^ On the other hand, when MSN‐SH(E) reacted with GSH alone (Figure 4B), relatively low amounts of GSSG were produced to give a GSH/GSSG ratio of ≈30 and ≈4 after reacting for 5 and 30 min, respectively. The generation of GSSG is known to be associated with the autoxidation of GSH (and the MPS groups on MSN‐SH(E)) by the molecular oxygen dissolved in solution, a process that mainly involves the deprotonation of thiol groups to form corresponding thiolate ions that react with molecular oxygen to generate reactive radical species.^[^ ^37^ ^]^ Since the p*Ka* (≈7.2) of the MPS groups on MSN‐SH(E) was lower than that (≈9.0) of GSH,^[^ ^38^, ^39^ ^]^ the oxidation of MPS groups should be faster than GSH, and the amount of GSSG generated in the presence of MSN‐SH(E) should be larger than that produced in the absence of MSN‐SH(E). As anticipated, the amounts of GSSG for the control groups with GSH alone or with B‐MSN were much lower than the group with MSN‐SH(E) (i.e., much higher GSH/GSSG ratios).

### 2.3 Effects of MSN‐SH on Permeation Enhancement

In addition to the mucoadhesive effect, the thiol groups on MSN‐SH(E) may play a role in enhancing the permeation of the nanovector. It has been shown that the GSH/GSSG ratio affects the opening of tight junctions in intestinal epithelia. A comparatively high level of GSH on the mucous membrane often results in the opening of an epithelial tight junction to further enhance permeation.^[^ ^40^ ^]^ It has been shown that the polymer‐attached cysteine thiol groups assisted drug permeation via interaction with GSSG to form disulfide bonds and release GSH, and that the increased level of GSH downregulated the activity of protein tyrosine phosphatase (PTPase) and upregulated protein tyrosine kinases (PTKs), leading to an enhanced degree of phosphorylation of tight junction proteins (e.g., occluding) to facilitate the opening of tight junctions.^[^ ^20^ ^]^ The same mechanism of permeation enhancement should also take place for MSN‐SH(E) capable of reacting and consuming more than 80% GSSG within 30 min to produce GSH efficiently via thiol/disulfide exchange (cf. Figure 4A).

**Figure 5:**
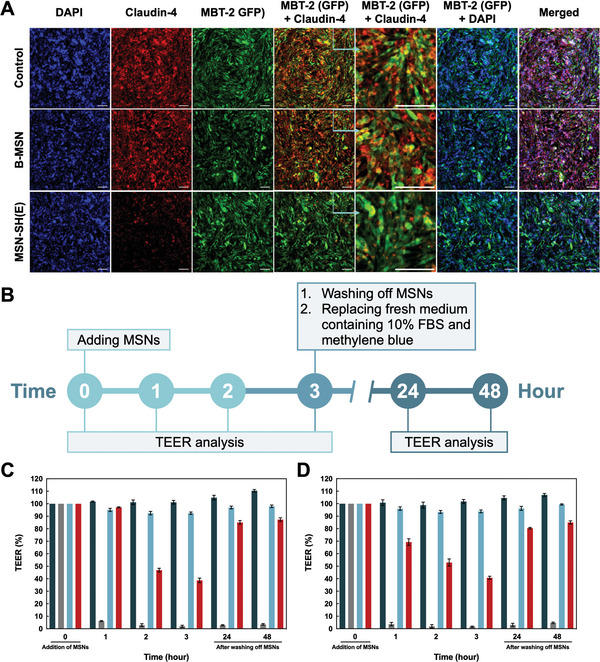
The role of claudin‐4 in tight junction opening triggered by two MSN nanoparticles on MBT‐2 (GFP) monolayer and enhanced permeation confirmed by TEER analysis. A) The CLSM images of claudin‐4 (red) and nuclei (blue) on MBT‐2 (GFP) (green) monolayers incubated with blank culture medium, B‐MSN and MSN‐SH(E) for 3 h. Scale bar = 100 µm. B) The timetable of TEER study. The relative TEER of C) MBT‐2 and D) Caco‐2 monolayers were co‐incubated with B‐MSN and MSN‐SH(E), respectively. TEER readings were taken at several time points (0, 1, 2, and 3 h) during nanoparticle incubation. Recovery of cell monolayer was verified after removal of nanoparticles and glutathione for 24 and 48 h. All the results are shown as mean ± SD, *n* = 3.

Alternatively, TPK Src protein plays an important role in opening tight junctions. The activation/phosphorylation of TPK Src can be triggered by interactions between thiomers and cysteine‐rich domain of membrane epidermal growth factor receptor (EGFR) and insulin‐like growth factor receptor (IGFR). This induces the redistribution of claudin‐4, resulting in the opening of the tight junctions.^[^ ^26^, ^41^ ^]^ To investigate the role of claudin‐4 in the MSN‐SH(E)‐mediated enhanced permeation, MBT‐2 (GFP) cells were treated with various MSNs and were observed by confocal microscopic system (CLSM). As shown in **Figure** **5**A, the claudin‐4 proteins were found evenly distributed in MBT‐2 (GFP) cell monolayers for both the control group and the B‐MSN‐treated groups, indicating a normal barrier integrity regulated closely by tight junctions, fragmental distribution of this protein was observed in the MSN‐SH(E)‐treated groups. The results implied that the opening of tight junctions may be related to the thiolated MSN, and therefore a transepithelial electrical resistance (TEER) analysis was carried out to confirm the phenomenon. Triton X‐100, a penetration agent for cells, was used as positive control in the measurements of TEER in Caco‐2 and MBT‐2 cells. As indicated in Figure 5B–D, MSN‐SH(E) induced a time‐dependent opening of tight junctions in both cells as evidenced by the significant reduction of resistance, whereas B‐MSN only triggered a minor drop of resistance, suggesting a lesser extent of permeation enhancement. Next, the effect of MSN‐ or Triton X‐100 on the recovery of barrier integrity of the cell was studied. Results showed that the values of TEER increased with time except for the Triton X‐100‐treated group. The values almost changed back to the initial ones for the B‐MSN‐treated groups, whereas ≈80–88% recovery was observed for the MSN‐SH(E) group. Treatment with MSN‐SH(E) improved both the apparent permeability coefficient (*P*_app_) and the transport enhancement ratio (*R*) of methylene blue through Caco‐2 and MBT‐2 cells by 4.25‐fold and 7.78‐fold, respectively (**Table** **1**). These results substantiated the permeation enhancement effect of MSN‐SH(E).

**Table 1:** Permeability of B‐MSN and MSN‐SH(E) encapsulating methylene blue. Apparent Permeability Coefficients (*P*_app_) of methylene blue and transport enhancement ratio (*R*) on MBT‐2 cell monolayer and Caco‐2 cell monolayer were calculated and listed below. All the results are shown as mean ± SD, *n*=3

Sample	Apparent permeability coefficient (*P*_app_ × 10^‐6^ [cm s^‐1^])		Transport enhancement ratio *R* = [*P*_app_(sample)/*P*_app_(control)]	
	MBT‐2 monolayer	Caco‐2 monolayer	MBT‐2 monolayer	Caco‐2 monolayer
Control	4.51 ± 1.71	9.36 ± 1.71	–	–
Triton X‐100	44.4 ± 1.12	60.80 ± 6.55	10.73 ± 3.60	6.67 ± 1.58
B‐MSN	9.73 ± 1.12	20.91 ± 2.96	2.34 ± 0.79	2.29 ± 0.58
MSN‐SH(E)	31.72 ± 2.81	38.43 ± 6.55	7.78 ± 3.25	4.25 ± 1.35

### 2.4 Immunoregulatory Effect of MSN‐SH(E) on Macrophage Polarization In Vitro

Silica‐based nanoparticles have been reported to have influential effects on the polarization of macrophages.^[^ ^42^ ^]^ The pro‐inflammatory M1 macrophage is characterized for the anti‐tumor properties, the ability in production interleukin 12 and 23 (IL‐12 and IL‐23), and the capacity in presenting antigens^[^ ^43^ ^]^ and activating T‐cell‐mediated anti‐tumor responses.^[^ ^44^ ^]^ Herein, the immunoregulatory effects of B‐MSN and MSN‐SH(E) on the polarization of M1/M2 subsets were explored. Treatments with (IFN‐*γ* + LPS) or IL‐4 were used to polarize macrophage (J774a.1) to M1‐like or M2‐like subsets, respectively. The polarization‐inducing cytokines (i.e., IFN‐*γ* + LPS or IL‐4) were subsequently removed by medium change before co‐incubation of various MSNs (B‐MSN and MSN‐SH(E)). The expression level of the induced M1 markers (NOS2, IL‐6, IL‐23, and TNF‐*α*) and M2 markers (ARG‐1, MRC‐1, and IL‐10) were analyzed by qPCR.

**Figure 6:**
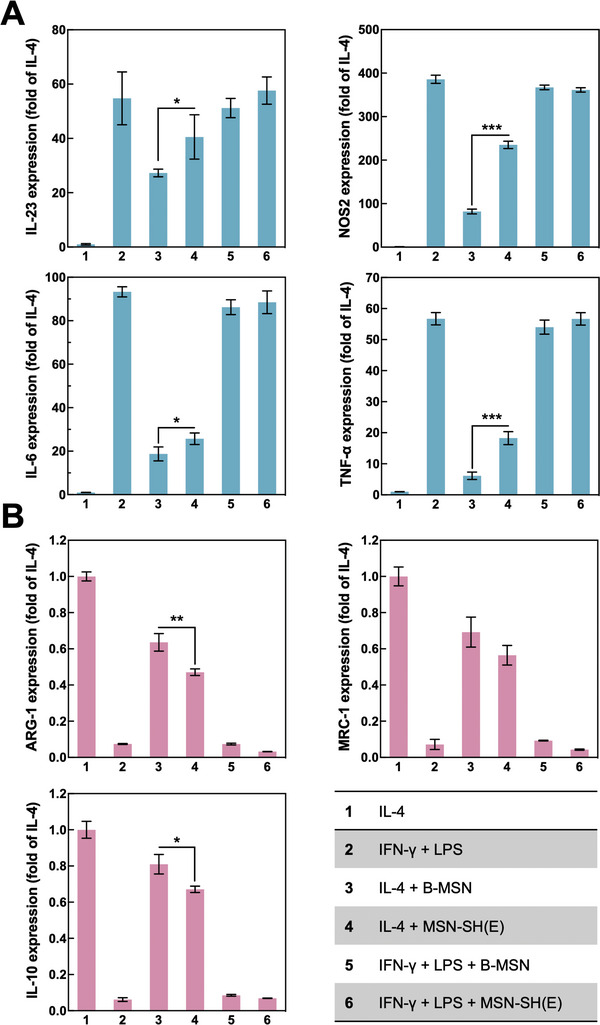
Effect of B‐MSN and MSN‐SH(E) on macrophage polarization. A) Pretreatment of IFN‐*γ* + LPS was used to polarize M0‐like cells to M1‐like macrophages. B) IL‐4 was used to polarize M2‐like cells. The expression of M1 or M2 markers was determined by RT‐qPCR. Cells were washed before addition of B‐MSN or MSN‐SH(E). Cell groups pre‐treated with IL‐4 or IFN‐*γ* + LPS served as normalized control groups (taking its mRNA expression level as 1) or experimental controls (its mRNA expression level was upregulated for M1‐like subset, whereas downregulated for M2‐like subsets), respectively. All the results are displayed as mean ± SD, *n* = 3 (*p < 0.05, **p < 0.01, and ***p < 0.001).

As depicted in **Figure** **6**A, both B‐MSN and MSN‐SH(E) induced comparable expression levels of M1 markers in IFN‐*γ* + LPS‐pretreated cells. However, B‐MSN and MSN‐SH(E) also promoted the expression levels of M1 markers in IL‐4 treated groups. We thus concluded that both B‐MSN and MSN‐SH(E) reduced the expression of M2 markers in M2‐like macrophages, and MSN‐SH(E) even outperformed B‐MSN in inhibiting M2 polarization. (Figure 6B) It has been intensively discussed^[^ ^45^ ^]^ that the regulation of M2‐like phenotype toward M1‐like phenotype is an emerging anti‐tumor strategy. Our observations confirm that MSN‐SH(E) holds the potential in reprogramming macrophages (M2 to M1), and therefore is a promising candidate for tumor immunotherapy.

Silica‐based nanoparticles were previously reported to elicit pro‐inflammatory responses and the production of reactive oxygen species (ROS) in macrophage RAW264.7.^[^ ^46^ ^]^ It has also been mentioned that surface modification of silica and other nanoparticles significantly influences the nanoparticle–cell interactions and facilitates cell uptake^[^ ^47^, ^48^ ^]^ In this study, we observed that the MPS groups modified on MSN‐SH(E) were capable of interacting with the cells and facilitating the reprogramming of M2 macrophages to M1‐like cells to enhance antitumor efficacy. Based on these findings, we thus conclude that MSH‐SH(E)s may be promising to target a tumor infiltrating M2‐like macrophage to achieve a superior anticancer immunotherapeutic strategy^[^ ^49^ ^]^ with enhanced efficacy.^[^ ^27^ ^]^

### 2.5 Anticancer Effects of MMC@MSN‐SH(E) in both In Vitro and In Vivo Models

The cytotoxicity of various formulations of MMC on bladder cancer cells was further evaluated. Groups of free MMC, MMC@B‐MSN, and MMC@MSN‐SH(E) (each treatment containing an equal amount of MMC, 0.125 mg mL^−1^) as well as MMC‐free control groups of B‐MSN and MSN‐SH(E) were incubated with MBT‐2 (GFP) cells for 2 h and washed with PBS every 2 h for 12 times in 24 h to mimic bladder washing and voiding of urine. As shown in Figure S3, Supporting Information, the cell viability of MMC@MSN‐SH(E) treatment (≈37.57%) was significantly lower than those for the groups of MMC (≈53.39%) and MMC@B‐MSN (≈52.78%). Since the cell viabilities of the MMC‐free groups were relatively high, the observed pronounced cytotoxicity effects of MMC@MSN‐SH(E) may be attributed to the extended release and prolonged residence of MMC. The attachment and drug release of the nanoparticles on the MBT‐2 (GFP) cells was further confirmed using R6G@B‐MSN and R6G@MSN‐SH(E). The fluorescence intensity of R6G in the cells treated with R6G@MSN‐SH(E) was found to be stronger than that treated with R6G@B‐MSN (Figure S4, Supporting Information), again demonstrating the enhanced cell attachment and R6G release of MSN‐SH(E) even after consecutive washing.

**Figure 7:**
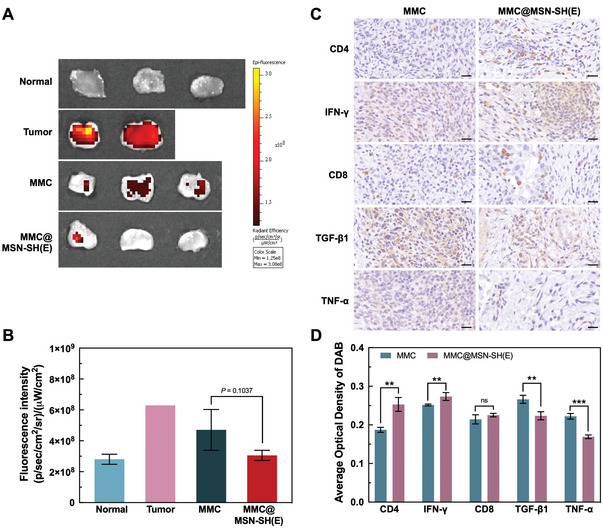
In vivo antitumor activity and immunomodulatory effect of MMC@MSN‐SH(E) nanoformulation. A) Bladders were dissected, opened longitudinally, and examined by IVIS. B) Quantification of fluorescence intensities for bladders from 4 animal groups to reveal the residual amounts of MBT‐2 cells (GFP) in the bladder. The fluorescence intensity of MBT‐2 (GFP) cancer cells was normalized to the tumor surface area. C) Representatives of tumor tissues taken from mice treated with MMC and MMC@MSN‐SH(E) were stained with CD4, IFN‐*γ*, CD8, TGF‐*β*1, and TNF‐*α*. Scale bar = 20 µm. D) A comparison of average optical density (AOD) between MMC and MMC@MSN‐SH(E)‐treated groups that were obtained by quantitative immunohistochemical analysis using ImageJ. All determinations were performed in triplicate and results were expressed as mean ± standard deviation (SD) (*p < 0.05 and **p < 0.01).

The anticancer effects of free MMC and MMC@MSN‐SH(E) on MBT‐2 (GFP) bladder tumor bearing mice are evaluated (Figure S5, Supporting Information). Schematic illustration of the experimental timeline is indicated in Figure S6, Supporting Information. It was found that the tumor group without any drug treatment exhibited the highest levels of fluorescence intensity than those treated with MMC or MMC@MSN‐SH(E), revealing the effective anticancer effect of MMC (**Figure** **7**A,B). Moreover, the group treated with MMC@MSN‐SH(E) showed significantly lower level (*p* = 0.1037) of fluorescence than that treated with free MMC, indicating enhanced effectiveness of MMC@MSN‐SH(E) nanoformulation in killing MBT‐2 cells and shrinking tumors.

### 2.6 Immunomodulatory Effect of MMC@MSN‐SH In Vivo

The immune responses mediated by free MMC and MMC@MSN‐SH(E) were examined by IHC staining. Results indicated that MMC@MSN‐SH(E) elicited a higher level of CD4^+^ T cells recruitment and IFN‐*γ* production (Figure 7C,D) than free MMC did. It was reported previously that MSN may induce the proliferation of lymphocytes and stimulate the secretion of cytokines, leading to the promotion of Th1 and Th2 immune responses and the enhancement of anticancer immunity.^[^ ^50^ ^]^ MMC@MSN‐SH(E)‐mediated upregulation of IFN‐*γ* may promote type I immunity and enhance antitumor ability. As observed in Figure 7C,D, tumors were significantly reduced after treating with MMC@MSN‐SH(E). It is also noticed that MMC@MSN‐SH(E)‐mediated increase of IFN‐*γ* production was accompanied by elevated recruitment of CD4^+^ T cells. On the other hand, CD8^+^ T cells, also IFN‐*γ*‐producing cells,^[^ ^51^ ^]^ did not exhibit in our study a significantly different infiltration in the tumor from those treated with free MMC.

The cause of increased recruitment of CD4^+^ T cells was further investigated by transwell assay. MSN‐SH(E) was co‐incubated with M0, M1‐ or M2‐like macrophages (J774a.1) in the upper chamber, and bladder cancer cells (MBT‐2) were incubated in the lower chamber. Results indicated that M1‐like cells co‐cultured with MSN‐SH(E) induced the highest level of IP‐10 (CXCL10) chemokine production in MBT‐2 cells than those co‐incubated with M0 and M2‐like cells (Figure S7, Supporting Information). It is known that IP‐10 can be induced by inflammatory cytokines, resulting in the recruitment of CD4^+^ T cells and macrophages.^[^ ^27^ ^]^ The increased production of IP‐10 in MBT‐2 cells mediated by MSN‐SH(E) and M1‐like cells could account for the enhanced recruitment of CD4^+^ T cells in vivo.

Additionally, treatment with MMC@MSN‐SH(E) decreased the levels of TNF‐*α* and TGF‐*β*1 compared with those of free MMC‐treated groups in vitro (Figure 7C,D). TGF‐*β*1, a cytokine released by M2‐like and TAM, has long been linked to immunosuppression^[^ ^52^ ^]^ and M2 polarization,^[^ ^53^ ^]^ promoting tumor development. However, TGF‐*β*1 signaling also suppresses tumor formation via inhibition of cell proliferation.^[^ ^54^ ^]^ Though TGF‐*β*1 plays a paradoxical role in cancer explored in recent years,^[^ ^55^ ^]^ as seen in our results, it is likely that MMC@MSN‐SH(E) downregulated more TGF‐*β*1 production and inhibited tumor formation as compared with the free MMC group. On the other hand, lower levels of TNF‐*α* observed in MMC@MSN‐SH(E)‐treated cells might imply decreased tumor aggressiveness and malignancy since high levels of TNF‐*α* are usually found in patients with malignant bladder tumors.^[^ ^56^ ^]^ Our in vitro studies revealed that MSN‐SH(E) may skew the macrophage toward M1‐like phenotype. Assumedly, MMC@MSN‐SH(E)‐assisted intravesical therapy may hold a great potential to elicit immunomodulatory effects in treating NMIB.

## 3 Conclusion 

The enhancement of MMC efficacy for NMIBC treatment has long been reviewed and developed for years; however, novel strategies remain required. A nanoformulation, MMC@MSN‐SH(E), with versatile utilities was fabricated herein. The thiol groups grafted on the MSN not only assisted its attachment onto the bladder mucosal layer, but also provided an enhanced permeation ability to efficiently deliver MMC into tumor tissues. In addition, MMC@MSN‐SH(E) showed stronger anti‐tumor responses and induced reprogramming of M2‐like macrophages to M1‐like phenotype than free MMC did in vitro. Intravesical delivery that avoids systemic circulation endorses the biosafety of MMC@MSN‐SH(E). It is believed that new opportunities can be enlightened by employing MMC@MSN‐SH(E) in treating NMIBC.

## 4 Experimental Section

### Materials

Benzylcetyldimethylammonium chloride (BCDAC), diethylene glycol hexadecyl ether (C_16_E_2_), fluorescein isothiocyanate (FITC), mitomycin C (MMC), Ellman's reagent (5,5′‐dithio‐bis‐(2‐benzoic acid), Rhodamine 6G (R6G) dye, methylene blue, L‐Glutathione reduced (GSH), L‐Glutathione oxidized (GSSG), L‐cysteine, formaldehyde, 4′,6‐diamidino‐2‐phenylindole (DAPI), bovine serum albumin (BSA), and lipopolysaccharide (LPS) were obtained from Sigma‐Aldrich (St Louis, MO, USA). MTT (3‐[4,5‐dimethylthiazol‐2yl]‐2,5‐diphenyl‐tetrazolium bromide) reagent was purchased from Cyrusbioscience (New Taipei City, TWN). Tetraethoxysilane (TEOS) was purchased from Acros Organics (Geel, BEL). 3‐Mercaptopropyltrimethoxysilane (MPTMS) and aminopropyltrimethoxysilane (APTMS) were purchased from Gelest (Morrisville, PA, USA). Recombinant murine IL‐4 and recombinant murine IFN‐*γ* were purchased from PeproTech (Cranbury, NJ, USA).

J774a.1 cells (mouse monocyte macrophage, ATCC TIB‐67) and Caco‐2 cells (human colorectal adenocarcinoma, ATCC HTB‐37) were ordered from Bioresource Collection and Research Center (BCRC) (Hsinchu, TWN). MBT‐2 cells, a type of murine bladder cancer cells with epithelial characteristics, were kindly provided by Dah‐Shyong Yu at Tri‐Service General Hospital (Taipei, TWN). Dulbecco's modified eagle medium (DMEM), RPMI‐1640 medium, and fetal bovine serum (FBS) were obtained from Thermo Fisher Scientific (Waltham, MA, USA). HyClone penicillin‐streptomycin 100× solution (P/S) was purchased from Cytiva (Washington D.C., USA). Anti‐CD4 antibody, anti‐IFN‐*γ* antibody, anti‐TGF‐*β*1 antibody, anti‐TNF‐*α* antibody, DAB (TA‐060‐QHSX and TA‐002‐QHCX), goat anti‐mouse IgG (H+L) secondary antibody (Alexa Fluor 555), and GSH/GSSG Ratio Detection Assay Kit II (Fluorometric‐Green) were acquired from Abcam (Cambridge, MA, USA). Anti‐CD8 antibody was obtained from Cell Signaling Technology (Danvers, MA, USA). Anti‐claudin‐4 antibody (A‐12) was purchased from Santa Cruz Biotechnology (Dallas, TX, USA). GAPDH, IL‐23, NOS2, IL‐6, TNF‐*α*, ARG‐1, IL‐10, MRC‐1, and IP‐10 primers (sequence details shown in Table S1, Supporting Information) were synthesized by Integrated DNA Technologies (IDT) (Coralville, Iowa, USA). Total RNA Isolation Kit (Blood/Cultured Cell/Fungus) was purchased from GeneDireX (Taoyuan, TWN). iScript cDNA Synthesis Kit and iQ SYBR Green Supermix kit were purchased from Bio‐Rad (Hercules, CA, USA).

Lentivirus plasmids including pMD.G, pCMV‐ΔR8.91, and pCMV‐Luc‐GFP‐2AA‐Puro were obtained from National RNAi Core Facility at Academia Sinica (Taipei, TWN). jetPRIME was a transfection reagent which was purchased from Polyplus (Bioparc, FRA). Puromycin was bought from InvivoGen (San Diego, CA, USA). Zoletil was purchased from Virbac Animal Health (Carros, FRA). Rompun was acquired from Bayer (Leverkusen, DEU).

### Apparatus

Varioskan LUX multimode Microplate reader was purchased from Thermo Fisher Scientific (Waltham, MA, USA). In vivo imaging system (IVIS, model Luminar II) was obtained from Caliper Life Sciences (Hopkinton, MA, USA). Millicell ERS‐2 Voltohmmeter was acquired from Merck (Darmstadt, Germany). Confocal microscope system (LSM780) was obtained from Zeiss (Jena, Germany). Real‐time PCR system (LightCycle 96 System) was obtained from Roche Molecular System, Inc. (Pleasanton, CA, USA). X‐ray diffractometer (XRD, 18MPX diffractometer) was obtained from MAC Science (Yokohama, Kanagawa, JPN). Adsorption analyzer (TriStar II Plus) was obtained from Micromeritics (Norcross, GA, USA). Field emission scanning electron microscope (FESEMs, JSM‐700F) and transmission electron microscope (TEM, JEM‐2010) were obtained from JEOL (Akishima, Tokyo, JPN). Nuclear magnetic resonance spectroscopy (NMR spectroscopy, Avance III HD 400 MHz) was obtained from Bruker (Billerica, MA, USA). Thermogravimetric analyzer (TGA/DSC 2‐HT) was obtained from Mettler‐Toledo (Columbus, OH, USA).

### Preparation and Characterization of Pure‐Silica and Functionalized MSNs

The hollow type MMT‐2 MSNs were synthesized by following the procedures reported previously.^[^ ^29^, ^30^ ^]^ BCDAC (0.74 g), C_16_E_2_ (0.26 g), 0.4 m NaOH (19 mL), and water (575 mL) were mixed and stirred in a polypropylene bottle. TEOS (5.98 mL) was then injected into the bottle at a rate of 7.5 mL h^−1^ under vigorous stirring, and the mixture was stirred at 35 °C for 2 h and subsequently aged at 90 °C for 24 h. The solid product was filtered, washed, and dried at 90 °C. To prepare the surfactant‐free MSNs, the as‐synthesized sample of MSNs (0.5 g) was stirred in the acidified ethanol (two drops of concentrated HCl solution in 50 mL ethanol) for 6 h and then filtered. The procedure was repeated three times to remove surfactants completely, and the sample of bare MSNs was dried at 80 °C and was designated as B‐MSN.

For the preparation of the functionalized MSNs with thiol groups on the external surface, the as‐synthesized sample of MSNs (0.5 g) was dried at 80 °C in vacuum for 12 h and was then poured and stirred in a toluene solution of MPTMS (8%, 50 mL) at 80 °C for 24 h in an argon atmosphere. The resulting solid was filtered, washed with toluene, then ethanol, and finally the remaining surfactants in the solid were repeatedly extracted by the acidified ethanol. The resulting surfactant‐free sample with MPS groups grafted on the external surface of MSNs was dried at 60 °C and was designated as MSN‐SH(E). Optionally, the interior mesopore surface of MSN‐SH(E) was further grafted by aminopropylsilyl groups following the same procedure for thiol modification except when using APTMS instead of MPTMS. The bifunctional sample (0.5 g) was then stirred in an ethanol solution (25 mL) of FITC (10 mg) at room temperature for 24 h, allowing the conjugation of FITC with aminopropylsilyl groups on the mesopores of MSN‐SH(E). The resulting solid was washed with ethanol and dried at 60 °C and was designated as MSN‐SH(E)/FITC(I). A sample with FITC conjugated on both external and mesopore surface was also prepared by first reacting B‐MSN with APTMS followed by conjugating with FITC based on the same procedures as aforementioned. The sample was designated as MSN‐FITC.

The pure‐silica and functionalized MSNs were characterized by multiple techniques including X‐ray diffraction (XRD), nitrogen physisorption, SEM, TEM, solid‐state ^29^Si MAS NMR, and thermogravimetric analysis (TGA). XRD patterns were obtained on a Mac Science 18MPX diffractometer using Cu K*α* radiation. Nitrogen physisorption isotherms were measured at 77 K using a Micromeritics TriStar II Plus instrument. SEM images were recorded with a field emission JEOL JSM‐7000F microscope operated at 10 kV. The SEM specimens were coated with Pt before measurements. TEM images were taken by using a JEOL JEM‐2010 microscope operated at 200 kV. Solid‐state ^29^Si MAS NMR spectra were measured on a Bruker Avance III 400 MHz NMR spectrometer using 4 mm MAS probe. TGA data were obtained using a Mettler‐Toledo TGA/DSC 2‐HT device.

### Preparation of MMC‐Loaded MSNs, its Release Profile and Cytotoxicity

The MMC‐loaded MSNs samples were prepared by wet impregnation. B‐MSN (2 mg) or MSN‐SH(E) (2 mg) was dispersed in a PBS solution of MMC (0.125 mg), and the mixture was stirred at 37 °C overnight to allow the solvent to be completely evaporated. The dried MMC‐loaded samples were designated as MMC@B‐MSN and MMC@MSN‐SH(E), respectively.

The in vitro release profile of MMC from MMC@MSN‐SHE(E) (2 mg mL^−1^) was determined in PBS (pH 7.4) at 37 °C. Aliquots of 0.1 mL were collected at specified periods, and the absorbance at 364 nm was measured using a Varioskan LUX multimode Microplate reader. These aliquots were brought back into the original reaction mixture after the measurements to prevent variation in volume. The concentration of MMC in the aliquots was determined from the measured values of absorbance using a calibration curve.

The cytotoxicity of free MMC and the MMC‐loaded MSNs was evaluated using MBT‐2 cells grown in RPMI‐1640 supplemented with 10% FBS and 1% P/S. Cells were incubated in a 96‐well microplate at 1.2 × 10^4^ cells per well in a humid atmosphere with 5% CO_2_ at 37 °C until a confluent monolayer was formed and samples including free MMC, MMC‐loaded MSNs (MMC@B‐MSN and MMC@MSN‐SH(E)), and MMC‐free MSNs (B‐MSN and MSN‐SH(E)) were then added into wells (*n* = 7), followed by 2 h incubation. The concentrations of MMC examined herein (for free MMC and MMC‐loaded MSNs) were 1, 0.75, 0.5, 0.25 and 0.125 mg mL^−1^, and the amounts of empty nanovectors (B‐MSN and MSN‐SH(E)) were 2 mg mL^−1^. Culture medium was changed every 2 h for a total of 24 h to imitate urine voiding. A control that used MBT‐2 cells without the addition of neither MMC nor MSN was also included. The MTT assay was used to determine the half‐maximal inhibitory concentration (IC_50_) of free MMC, MMC‐loaded MSNs (MMC@B‐MSN and MMC@MSN‐SH(E)) against MBT‐2 cells. All determinations were performed in septuplicate and results were expressed as mean ± standard deviation (SD).

### Mucoadhesive Effect of MSN‐SH on Porcine Bladder

Fresh porcine bladders were obtained from a local slaughterhouse that were carefully cleaned and cut into 1 cm^2^. Various types of MSNs including MSN‐SH(E)/FITC(I), MSN‐FITC, R6G‐loaded MSN‐SH(E) (designated as R6G@MSN‐SH(E)) and R6G‐loaded B‐MSN (designated as R6G‐B‐MSN), were dispersed in PBS (2 mg mL^−1^) and homogeneously applied onto the surface of porcine bladders mucosa for 2 h, followed by washing with PBS (3 mL) for five times. The R6G‐loaded samples were prepared by impregnating R6G (1 mg) into B‐MSN (2 mg) or MSN‐SH(E) (2 mg) by following similar procedure for loading MMC into the two MSNs (cf. Section 2.4) The residual fluorescence was measured by IVIS Luminar II In Vivo Imaging System. The fluorescence intensity was expressed as the mean ± SD while at least three independent experiments were performed.

### Quantifying and Visualizing the Permeability of Cell Monolayers

Alterations in the GSH/GSSG ratio mediated by MSN‐SH(E) were detected by GSH/GSSG Ratio Detection Assay Kit II. B‐MSN (2 mg mL^−1^) or MSN‐SH(E) (2 mg mL^−1^) was allowed to react with either 45 µm GSSG or 45 µm GSH at 37 °C to simulate the human tissue environment.^[^ ^57^ ^]^ After incubation, the MSN sample was removed by centrifugation (16 000 rcf) before acquisition of fluorescence intensity for each sample. The level of GSH and total glutathione (GSH+GSSG) was measured according to the manufacturer's instructions at excitation/emission wavelengths of 490/520 nm. The average GSH/GSSG ratio was expressed as the mean ± SD from at least three independent experiments (*n* = 3).

The effect of thiomer (i.e., MSN‐SH(E)) on the membrane distribution of claudin‐4, expressed mainly at the tight junction, was investigated as described in the following: after seeding and serum starvation for 12 h, green fluorescent protein (GFP)‐expressing MBT‐2 (abbreviated as MBT‐2 (GFP)) monolayers were subjected to incubate with RPMI‐1640 containing B‐MSN (2 mg mL^−1^) and MSN‐SH(E) (2 mg mL^−1^), respectively, for 3 h at 37 °C. The cell monolayers were subsequently washed with PBS for 3 times and fixed in 4% formaldehyde for 30 min at room temperature. Cells were then permeabilized in 0.2% Triton X‐100 for 10 min. After reducing nonspecific binding with 5% bovine serum albumin (BSA), cell monolayers were sequentially incubated with mouse anti‐claudin‐4 antibody (1:100) at 4 °C overnight, and goat anti mouse IgG (H+L) secondary antibody (Alexa Fluor 555) (1:300) for 1 h at room temperature. Finally, cell monolayers were stained with DAPI for the nucleus and observed under a confocal microscopic system (CLSM).

The TEER of a cellular monolayer (in ohms, Ω) is a quantitative measurement of the barrier integrity and permeability.^[^ ^22^, ^58^, ^59^ ^]^ The monolayers of Caco‐2 or MBT‐2 cells for permeability measurement were herein used, where cells were seeded onto Millicell hanging cell culture insert with a pore size of 0.4 µm used in a 24 well plate, and cultured in Dulbecco's modified eagle medium (DMEM) for Caco‐2 and RPMI‐1640 for MBT‐2, respectively. The epithelial barrier completeness of cells was measured using Millicell ERS‐2 Voltohmmeter. To ensure intact cell monolayers with well‐established tight junctions, an initial TEER of 300–500 Ω·cm^2^ were employed for Caco‐2 monolayer, whereas TEER of 600–800 Ω·cm^2^ were with MBT‐2 monolayer.^[^ ^22^, ^58^, ^59^ ^]^

Cell monolayers in the donor chamber were incubated with B‐MSN (2 mg mL^−1^) or MSN‐SH(E) (2 mg) suspended in serum‐free medium. Also, Triton X‐100 (4% m v^−1^) was used as a positive control. After 3 h incubation, the MSNs sample and Triton X‐100 were carefully removed from the donor chamber, and the serum‐free medium was replaced with a fresh one containing 10% FBS and methylene blue (20 mg mL^−1^). Transwell inserts were continuously incubated in a CO_2_ incubator (5% CO_2_) at 37 °C. TEER readings were scheduled to acquire at 0, 1, 2, 3, 24 and 48 h, respectively, using an aqueous solution of methylene blue (20 mg mL^−1^) as the control. The absorbance of methylene blue at 665 nm at the receiver chamber was recorded every 30 min. The amount of methylene blue was determined using the calibration curve.

Apparent permeability coefficient (*P*_app_) of methylene blue was calculated based on the following formula:

(1) $$P_{app}=\frac{Q}{A\times c\times t}$$

where *Q* is the amount of methylene blue (in mg) permeated the monolayer, *A* is the diffusion area 0.3 cm^2^, *c* is the initial concentration (in mg mL^−1^) of methylene blue in the donor chamber, and *t* is the time (in second) used in this study. Further, transport enhancement ration (*R*) was calculated based on the following formula:

(2) $$R=\left[P_{\mathrm{app}}\left(\mathrm{sample}\right)/P_{\mathrm{app}}\left(\mathrm{control}\right)\right]$$

### Study of Regulatory Effects of MSN‐SH(E) on M1‐Like and M2‐Like Macrophages

J774a.1 cells were chosen herein to verify the regulatory effect of MSN‐SH(E) on the polarization of M1‐like and M2‐like phenotypes. Macrophages (M0, 2 × 10^6^ cells well^−1^ in a 24‐well plate, *n* = 3) were pre‐polarized to M1‐like or M2‐like subtypes via stimulation of IFN‐*γ* (20 ng mL^−1^) + LPS (10 ng mL^−1^) or IL‐4 (20 ng mL^−1^) for 24 h, respectively. B‐MSN (2 mg mL^−1^) or MSN‐SH(E) (2 mg mL^−1^) was subsequently co‐incubated with M1‐like or M2‐like cells for 2 h, followed by a medium change to remove the MSNs.

The secretion of IP‐10 (CXCL10) from MBT‐2 cells was measured by transwell plates, in which pre‐polarized M1‐like and M2‐like macrophages (6.3 × 10^5^ cells insert^−1^) were cultured in the donor chamber, while MBT‐2 cells (6 × 10^4^ cells well^−1^ in a 24‐well plate) were cultured in the accepter chamber. B‐MSN (2 mg mL^−1^) or MSN‐SH(E) (2 mg mL^−1^) were subsequently co‐cultured with M1‐like or M2‐like cells for 2 h, followed by a medium change of donor chamber to remove the MSNs, and an incubation for a further 24 h. At last, cells were collected, and total RNAs of MBT‐2 cells were extracted using a Total RNA isolation kit, followed by reverse transcription to cDNA using iScript cDNA Synthesis Kit. Gene expression level was quantified using iQ SYBR Green Supermix kit, and the primer sequences used herein are shown in Table S1, Supporting Information. The expression levels of GAPDH, IL‐23, NOS2, IL‐6, TNF‐*α*, ARG‐1, IL‐10, MRC‐1, and IP‐10 were determined by quantitative PCR (qPCR) using LightCycler 96 System, and the expression level of GAPDH was regarded as an endogenous control. The data were expressed as the mean ± SD (3 independent experiments were performed).

### Animal Study Using an Established Orthotopic Mouse MBT‐2 (GFP) Bladder Tumor Model

To establish orthotopic bladder cancer models, 6‐week‐old female C3H/HeNCrlBltw mice (with body weight of ≈20 g) were purchased from BioLASCO Taiwan Co., Ltd. (Taipei, TWN). All animal experiments were reviewed and approved by the Institutional Animal Care and Use Committee (IACUC) of Taichung General Hospital Laboratory Animal Center (La‐1091713). MBT‐2 (GFP) cells were used for implanting tumors to achieve non‐invasive bladder tumor in mice. To evaluate tumors within mice and for the better monitoring tumor growth and observing the response to various treatments, non‐invasive in vivo imaging was performed. Moreover, MBT‐2 cells were transfected using jetPRIME transfection reagent (Polyplus) with Lentivirus vector encoding GFP (Figure S2, Supporting Information, provided by National RNAi Core Facility at Academia Sinica) to establish a MBT‐2 (GFP) subclone. The excitation and emission wavelengths of GFP were 480 and 510 nm, respectively. Both MBT‐2 and MBT‐2 (GFP) cells were able to form tumors in the bladder.

MBT‐2 (GFP) cells were subsequently used to establish the orthotopic mouse bladder tumor model as described previously.^[^ ^60^, ^61^ ^]^ In brief, the mice were anesthetized with Zoletil (20 µL) and Rompun (10 µL) in 200 µL PBS intraperitoneally. The dosage was designed to keep mice sedated for about 2 h. Twenty‐four‐gauge (24‐G) intravenous cannula was placed into the urethra. Residual urine in the urinary bladder was drained out. The bladder was instilled with 0.1 mL of 0.1 N HCl for 15 s and then washed with 0.1 mL PBS. The same procedure was repeated with 0.1 mL of 0.1 n NaOH. The disruption of bladder mucosa facilitated the attachment of cancer cells. A suspension of MBT‐2 (GFP) cells (0.1 mL, 5 × 10^6^ cells mL^−1^) was installed into the bladder and was allowed to keep for 2 h. The presence of tumors in the bladder was confirmed by IVIS on the seventh day after implantation.

Four different groups of mice were evaluated, including: 1) control group without tumor; 2) orthotopic MBT‐2 (GFP) bladder tumor‐bearing group without treatment; 3) orthotopic MBT‐2 (GFP) bladder tumor‐bearing group treated with free MMC by infusion of 100 µL sterile solution of MMC (0.125 mg mL^−1^); and 4) orthotopic MBT‐2 (GFP) bladder tumor‐bearing group treated with MMC@MSN‐SH(E) by infusion of 100 µL sterile solution of MMC@MSN‐SH(E) (2 mg MSN‐SH(E) mL^−1^, corresponding to 0.125 mg MMC mL^−1^). The treatment was performed on day 9 and day 11.

Mice treated with free MMC and MMC@MSN‐SH(E) were anesthetized via intraperitoneal injection and catheterized with a 24‐G intravenous cannula. Solutions (0.1 mL) of MMC and MMC@MSN‐SH(E) were instilled with cannula and clumped for 15 s to keep MMC and MMC@MSN‐SH(E) to stay in the bladders of the mice. The solutions were retained in the bladders for 2 h and were voided after recovery from anesthesia. The treated mice were sacrificed humanely on the third day after the second course of treatment (on Day 14). Bladders were taken out and dissected longitudinally for analysis. The weight of bladders and gross appearance were recorded. The fluorescence of GFP was measured by the IVIS (Ex/Em: 480/510 nm). For further histological analysis, 5 µm‐thick unstained slides were prepared from the bladder tissues treated with free MMC or MMC@MSN‐SH(E).

Immunohistochemical staining (IHC) with rabbit antibodies to CD4^+^ T cells (1:300), CD8^+^ T cell antibody (1:200), IFN‐*γ* (1:200), TGF‐*β* beta 1 (1:100), and TNF‐*α* (1:100) were performed using an ABC (street(avidin)‐biotin complex) method with a DAB chromogen.^[^ ^62^ ^]^ Three complete and non‐overlapping regions of interest were randomly selected, and images were captured from an optical microscope with a magnification factor of ×300 times. The color intensity of tumor cells staining was categorized into three groups: weak, intermediate, and strong. The obtained IHC images were subjected to the quantitative analysis of the protein expression by measuring the optical density (OD) using the ImageJ software, and the average optical density (AOD = IOD/Area) was used as the evaluation standard.

### Statistical Analysis

Statistical data analysis was performed using Student *t*‐test. Statistical significance was assigned at the level of *p* ≤ 0.05. All values were expressed as the mean ± SD. The samples/animals were allocated to experimental groups and processed randomly.

## Conflict of Interest

The authors declare no conflict of interest.

## Author Contributions

C.C.C., Y.C.F., Y.Y.K., and Y.C.L. contributed equally to this work. C.M.Y.’s group was responsible for the synthesis, functionalization and characterization of MSNs; J.A.H.’s group helped with surface modification of MSNs, and was responsible for in vitro and in vivo studies with L.C.W.’s group. X.H.W. provided technical help for in vivo imaging (IVIS). All authors participated in the writing of the manuscript. All authors discussed the results and implications and commented on the manuscript at all stages. All authors read and approved the final manuscript.

## Ethics Statement

All animal experiments conducted in the current study were performed in compliance with the NHMRC Taiwan Code of Practice for the care and use of animals for scientific purposes, and approved by the Institutional Animal Care and Use Committee (IACUC) of Taichung Veterans General Hospital.

## Supporting information

Supporting Information

## Data Availability

The data that support the findings of this study are available from the corresponding author upon reasonable request.
