# Supplementary material for: Thiolated Mesoporous Silica Nanoparticles as an Immunoadjuvant to Enhance Efficacy of Intravesical Chemotherapy for Bladder Cancer
Source: Adv Sci (Weinh). 2023 Jan 13;10(7):2204643. doi: 10.1002/advs.202204643 (PMC9982584; doi:10.1002/advs.202204643)
Supplement: Supplementary file 1 — Supporting Information [file ADVS-10-2204643-s001.pdf]

ADVANCED SCIENCE
Open Access

Supporting Information

for *Adv. Sci.*, DOI 10.1002/advs.202204643

Thiolated Mesoporous Silica Nanoparticles as an Immunoadjuvant to Enhance Efficacy of Intravesical Chemotherapy for Bladder Cancer

Cheng-Che Chen, Yu-Chen Fa, Yen-Yu Kuo, Yi-Chun Liu, Chih-Yu Lin, Xin-Hui Wang, Yu-Huan Lu, Yu-Han Chiang, Chia-Min Yang, Li-Chen Wu* and Ja-an Annie Ho**

Supporting Information

Thiolated Mesoporous Silica Nanoparticles as an Immunoadjuvant to Enhance Efficacy of Intravesical Chemotherapy for Bladder Cancer

Cheng-Che Chen[1,2†], Yu-Chen Fa[1†], Yen-Yu Kuo[3†], Yi-Chun Liu[1†], Chih-Yu Lin[3], Xin-Hui Wang[4], Yu-Huan Lu[3], Yu-Han Chiang[5], Chia-Min Yang[3,6*], Li-Chen Wu[7*] and Ja-an Annie Ho[1,5,8,9*]

[1]BioAnalytical Chemistry and Nanobiomedicine Laboratory, Department of Biochemical Science and Technology, National Taiwan University, Taipei, 10617, Taiwan
[2]Department of Urology, Taichung Veterans General Hospital, Taichung, 40705, Taiwan
[3]Department of Chemistry, National Tsing Hua University, Hsinchu, 300044, Taiwan
[4]Instrumentation Center, National Taiwan University, Taipei, 10617, Taiwan
[5]Department of Chemistry, National Taiwan University, Taipei, 10617, Taiwan
[6]Frontier Research Center on Fundamental and Applied Sciences of Matters, National Tsing Hua University, Hsinchu, 300044, Taiwan
[7]Department of Applied Chemistry, National Chi Nan University, Puli, Nantou, 54561, Taiwan
[8]Center for Emerging Materials and Advance Devices, National Taiwan University, Taipei 10617, Taiwan
[9]Center for Biotechnology, National Taiwan University, Taipei, 10617, Taiwan

Table of Contents

1. Sequences details of primers for GAPDH, NOS2, TNF-α, IL-23, IL-6, ARG-1, MRC-1, IL-10....S3

2. Measurements of p*Ka*, stability of the MPS groups and quantification of the thiol groups on the MSN-SH(E)....S4

3. Schematic illustration of the pLenti-Glll-CMV-GFP-2A-Puro vector S8

4. Cytotoxic effect of various formulations of MMC on bladder cancer cells. S9

5. Cell attachment effect and R6G release from the nanoparticles. S10

6. Anticancer effects of free MMC and MMC@MSN-SH(E) on MBT-2 (GFP) cells. S11

7. Schematic illustration of the experimental timeline....S12

8. Expression of IP-10 (CXCL10) induced by B-MSN and MSN-SH(E) S13

Table:

Table. S1. Nucleotide sequences of specific primers. S3

Figure:

Figure S1. The p*Ka* and stability of the thiol groups on MSN-SH(E). S7

Figure S2. Construction of MBT-2 (GFP) cells expressing the GFP gene.. .... S8

Figure S3. Cell viability of MBT-2 cells treated with various concentration of free mitomycin C. S9

Figure S4. Cell attachment effect of MSN-SH(E) verified by fluorescence microscope. S10

Figure S5. Cell viability of MBT-2 cells treated with B-MSN, MSN-SH(E) and free mitomycin C. S11

Figure S6. Schematic of experimental timeline for the animal study. S12

Figure S7. IP-10 (CXCL10) expression induced by B-MSN and MSN-SH(E). . S13

1. Sequences details of primers for GAPDH, NOS2, TNF-α, IL-23, IL-6, ARG-1, MRC-1, IL-10

Table S1. Nucleotide sequences of specific primers.

Target Genes	Sequences (5’ to 3’)
GAPDH	F: GCAAA TTCAACGGCACAG<br>R: CACCAGTAGACTCCACGAC
NOS2	F: GAGACAGGGAAGTCTGAAGCAC<br>R: CCAGCAGTAGTTGCTCCTCTTC
TNF-α	F: GGTGCCTATGTCTCAGCCTCTT<br>R: GCCATAGAACTGATGAGAGGGAG
IL-23	F: AGCAACTTCACACCTCCCTAC<br>R: ACTGCTGACTAGAACTCAGGC
IL-6	F: ACGGCCTTCCCTACTTCACA<br>R: CATTTCCACGATTTCCCAGA
ARG-1	F: AAGAATGGAAGAGTCAGTGTGG<br>R: GGGAGTGTTGATGTCAGTGTG
MRC-1	F: GTTCACCTGGAGTGATGGTTCTC<br>R: AGGACATGCCAGGGTCACCTTT
IL-10	F: AGCCGGGAAGACAATAACT<br>R: AGGAGTCGGTTACCAGTATC
IP-10	F: ATCATCCCTGCGAGCCTATCCT<br>R: GACCTTTTTTGGCTAAACGCTTTC

2. Measurements of pKa, stability of the MPS groups and quantification of the thiol groups on the MSN-SH(E)

The pKa of the MPS (mercaptopropylsilyl, abbreviated as R-SH) groups on the external surface of MSN-SH(E) was determined by a spectrophotometric method originally proposed by Benesch and Benesch.[1] In brief, MSN-SH(E) (0.1 mg mL^{-1}) was dispersed in an aqueous solution of 0.001 м HCl and 0.1 м NaCl (for maintaining ionic strength). The aqueous solution of 0.01% NaOH was then added dropwise into the MSN-SH(E)-containing mixture under vigorous stirring to reach designated pH value. Concurrently the absorbance of the solution at 242 nm was measured by Varioskan LUX multimode Microplate reader. The pKa could be obtained by plotting pH versus $-log[(A_{max} - A_i)/A_i]$, where A_{max} is the maximum value of absorbance measured at the highest pH and Ai is the absorbance observed for a measurement i.[2]

The stability of R-SH groups on MSN-SH(E) against oxidation at different pH was also investigated. The dispersions of MSN-SH(E) in buffered aqueous solutions (0.5 mg mL^{-1}) at pH 4.0, 6.0 or 7.2 were prepared using acetate buffer (pH 4.0, 50 mм), phosphate buffer (pH 6.0, 50 mм), or Tris buffer (pH 7.2, 50 mм). The dispersions were continuously agitated at 37°C for various periods of time (0, 30, 60, 90, 120 or 180 min) before adding HCl (1.0 м, 10 μL) to stop further reaction. The amount of the remaining R-SH groups in the tested samples were quantified using Ellman's reagent according to the manufacturer protocol.[3] The absorbance of L-cysteine at 412 nm versus concentration was plotted as a calibration curve for thiol quantification.

The formation of disulfide bond requires conversion of two thiol groups, one of which first deprotonated to the corresponding thiolate anion to serve as nucleophile to attack the other. The increase in solution alkalinity facilitates the deprotonation of thiol groups to form the corresponding thiolate anions. In our case, just as other thiol

groups,[4-6] the MPS (abbreviated as R-SH) groups on the external surface of MSN-SH(E) exhibited nearly no absorption at around 242 nm while the corresponding thiolate R−S⁻ showed an intense absorption maximum at the wavelength. Taking advantage of such phenomena, we were able to determine the pKa of the thiol groups on MNS-SH(E) by measuring the absorbance of the solution (242 nm) at different pH. **Figure S1A** is essentially a titration curve of MSN-SH(E). The absorbance at 242 nm (A_i) was zero when the pH of solution was below 5.0, indicating the thiol groups were nearly exclusively in protonated form (R-SH). While further increasing solution pH, A_i was consequently elevated in value, attributing to fractional deprotonation of the thiol groups. A maximum value of A_i (A_{max}) was observed as pH was higher than 10.0. Since the absorbance at 242 nm is proportional to the percentage of R−S⁻ groups present in the sample, the ratio of $(A_{max} - A_i)/A_i$ is equal to the ratio of $([RSH]_{total} - [RS]_i)/[RS]_i$, or $[RSH]_i/[RS]_i$. A linear plot of pH versus $-log[(A_{max} - A_i)/A_i]$ is thus obtained **(Figure S1B)**, and the pKa of the MPS groups on MSN-SH(E) was found to be 7.2, which is significantly lower than the typical values (~8-9) for thiol groups.[7] It is known that the pKa measures the tendency of deprotonation of a thiol group that may be affected by its surrounding chemical environment.[8-9] The relatively low pKa of the MPS groups may be associated with the presence of the nearby surface silanol (Si-OH) groups with an isoelectric point of around pH 2.[10] The deprotonation of silanol groups at a pH higher than 2 leads to a negatively charged surface, which may further interact with the MPS groups on MSN-SH(E), leading to a decreased pKa. It is noted that a reduced pKa of a thiol group is beneficial for the formation of disulfide bonds.[11]

The stability of the MPS groups on MSN-SH(E) against possible oxidation by molecular oxygen dissolved in solutions was also investigated at solution pHs of 4.0,

6.0 and 7.4 by measuring the remaining thiol groups after designated periods of time using Ellman's reagent. As expected, almost all the MPS groups on MSN-SH(E) remained after 180 min at pH 4.0, indicating that the groups are stable at low pH. At higher pH, however, a significant fraction of thiol groups was oxidized, presumably to form intra-particle or inter-particle disulfides. As shown in **Figure S1C**, around 64% and 41% of the MPS groups originally present on MSN-SH(E) remained at pH 6.0 and 7.2, respectively**.** The amount of the MPS groups on the MSN-SH(E) was determined to be 2.20±0.07 mmol g^{-1}.

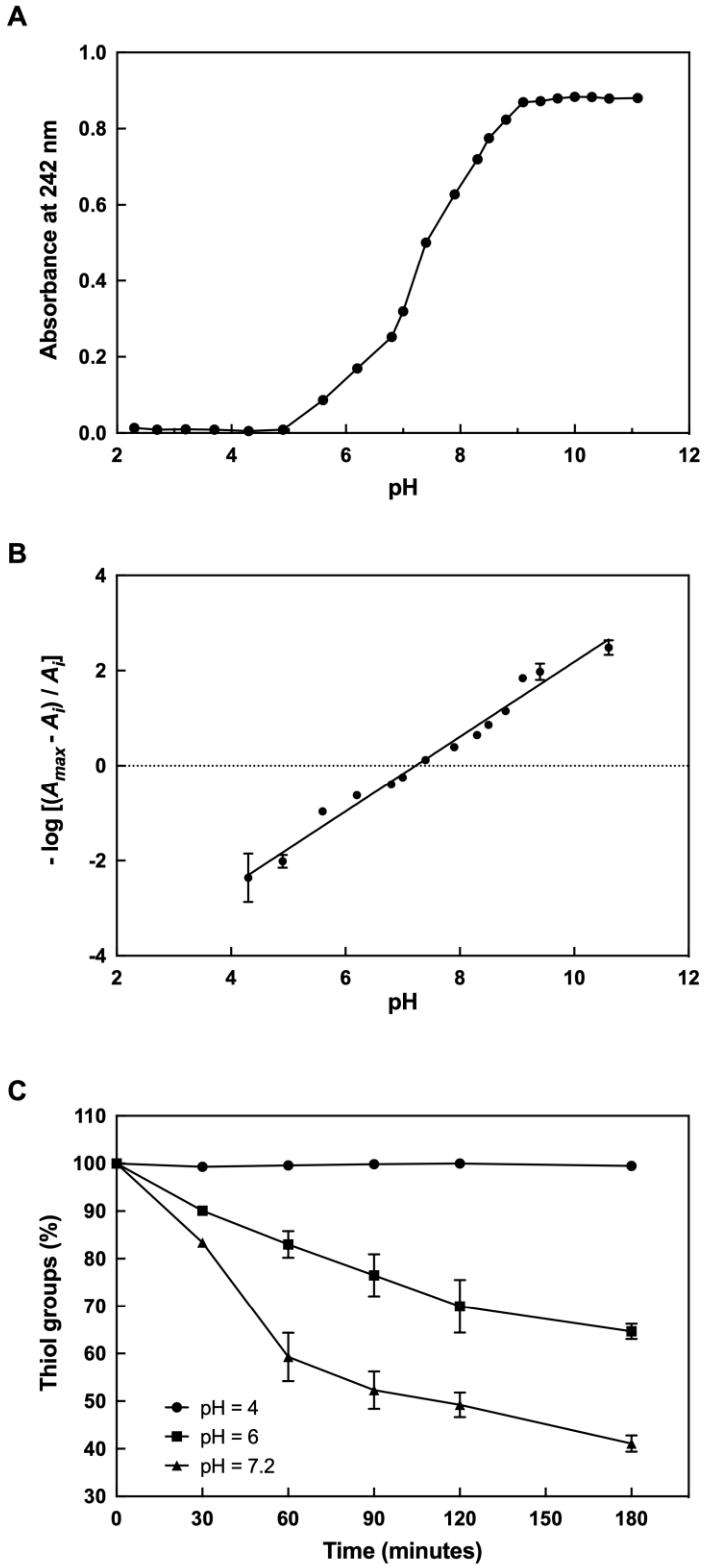

Figure S1. The p*Ka* and stability of the thiol groups on MSN-SH(E). (A) Absorbance at 242 nm as a function of pH for MSN-SH(E). (B) A plot of $-\log[(A_{max} - A_i)/A_i]$ *vs.* pH. (C) Stability of the thiol groups on MSN-SH(E) against oxidation at 37°C at pH 4.0 (●), 6.0 (■) and 7.2 (▲). All the results are shown as mean ± SD, n=3.

3. **Schematic illustration of the pLenti-GlIl-CMV-GFP-2A-Puro vector**

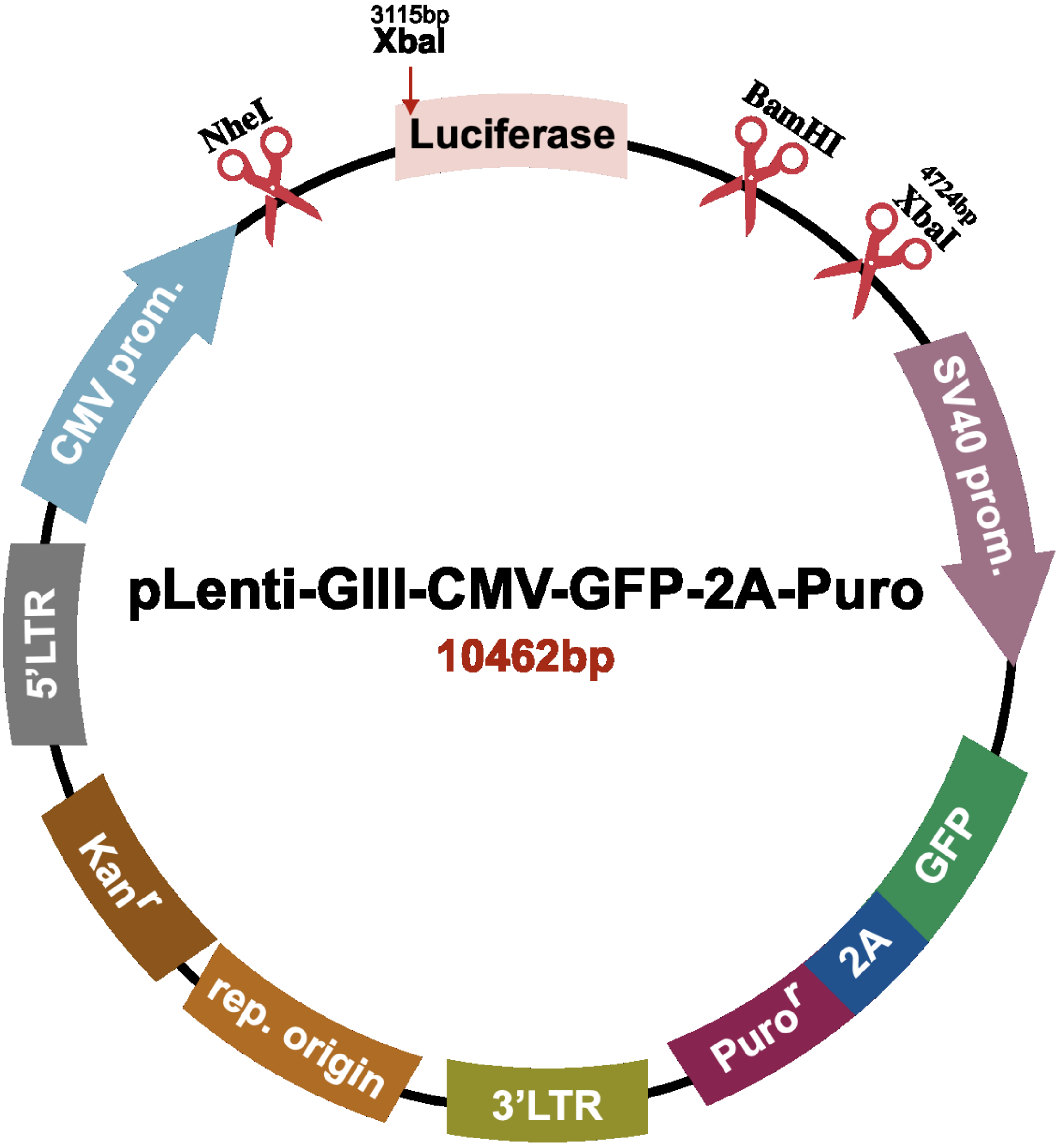

Figure S2. Construction of MBT-2 (GFP) cells expressing the GFP gene. The lentivirus shuttle vectorpLenti-GIII-CMV-GFP-2A-Puro was engineered to express luciferase and GFP expression cassette under the SV40 promoter. A three-plasmid-based lentiviral expression system was co-transfected with pMD.G (envelope plasmid), pCMV-ΔR8.91 (packaging plasmid) and pCMV-Luc-GFP-2AA-Puro.

4. Cytotoxic effect of various formulations of MMC on bladder cancer cells.

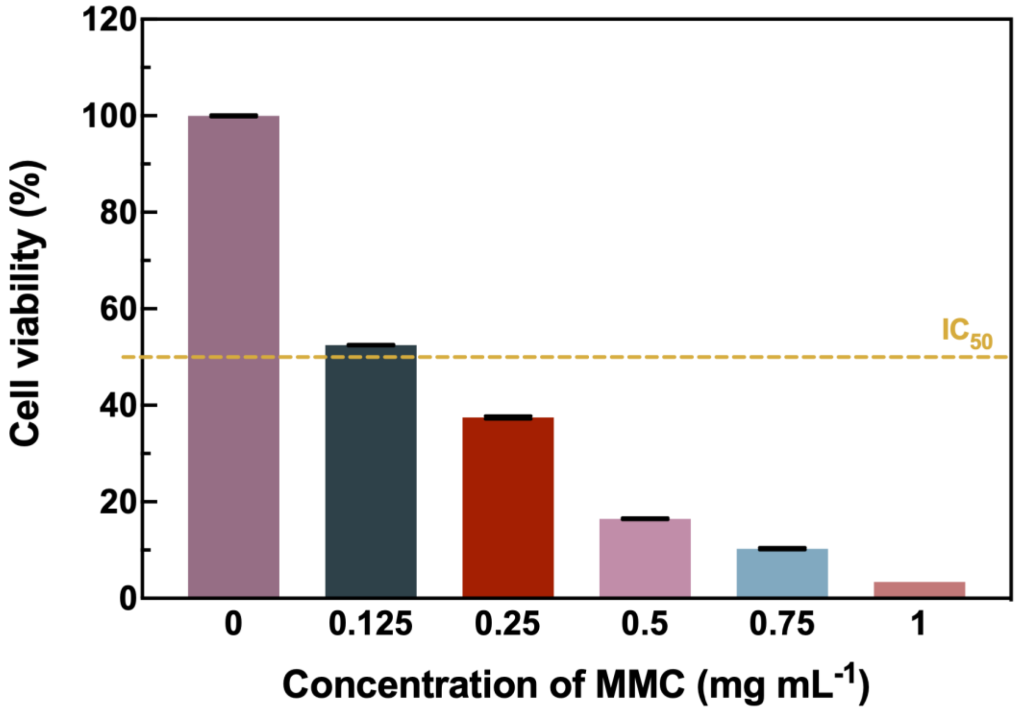

Figure S3. Cell viability of MBT-2 cells treated with various concentration of free mitomycin C. Cell medium was changed every 2 h during a 24 h period. Cell viability was determined by MTT assay. The IC_{50} of free MMC is determined to be 0.125 mg mL^{-1}. All the results are shown as mean ± SD, n=5.

5. Cell attachment effect and R6G release from the nanoparticles.

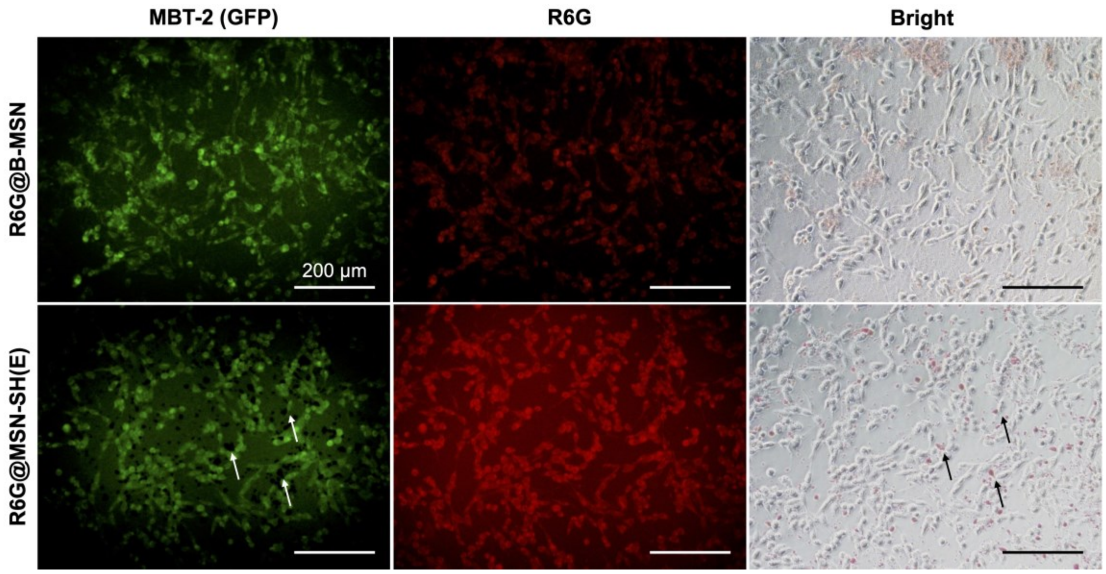

Figure S4. Cell attachment effect of MSN-SH(E) verified by fluorescence microscope. MSN-SH(E) demonstrated better cell attachment on MBT-2 (GFP) than B-MSN after consecutive washing, as indicated by the stronger fluorescent intensity of R6G released from R6G@MSN-SH(E). The arrow indicated the existence of nanoparticles. Scale bar=200 μm.

6. Anticancer effects of free MMC and MMC@MSN-SH(E) on MBT-2 (GFP) cells.

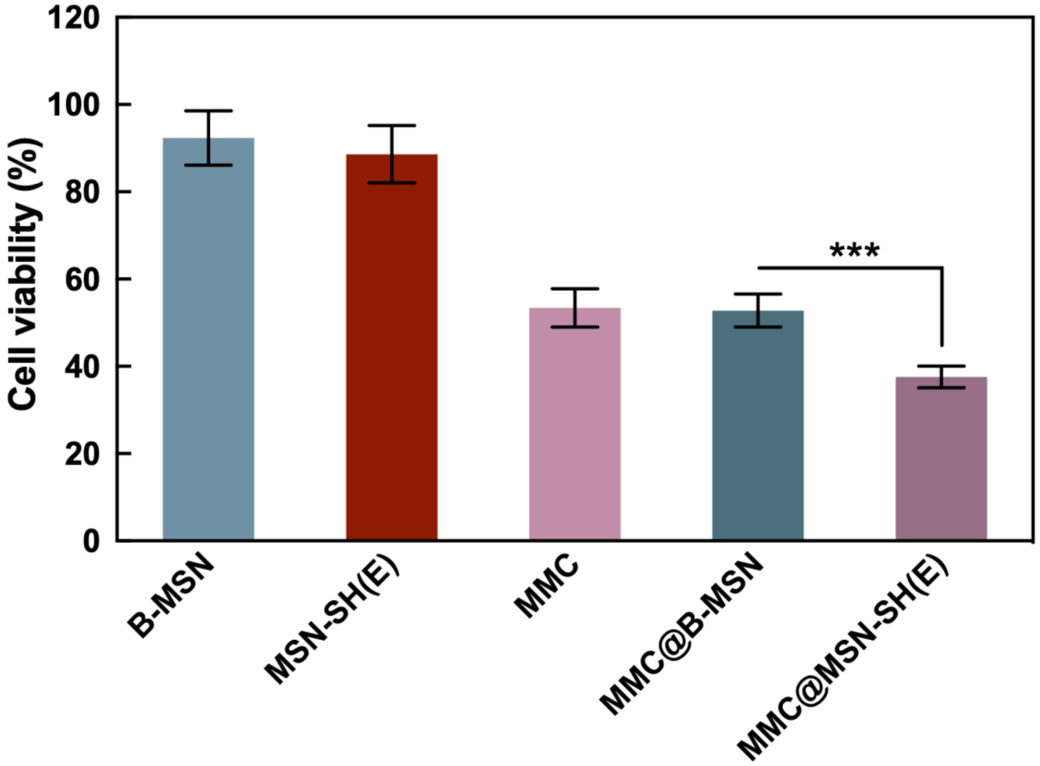

Figure S5. Cell viability of MBT-2 cells treated with B-MSN, MSN-SH(E) and free mitomycin C. The treated concentration of MMC was 0.125 mg mL^{-1}. Medium was changed every 2 h during 24 h period. Cell viability was determined by MTT assay. All the results are shown as mean ± SD, n=3. (***$P < 0.001$).

7. Schematic illustration of the experimental timeline

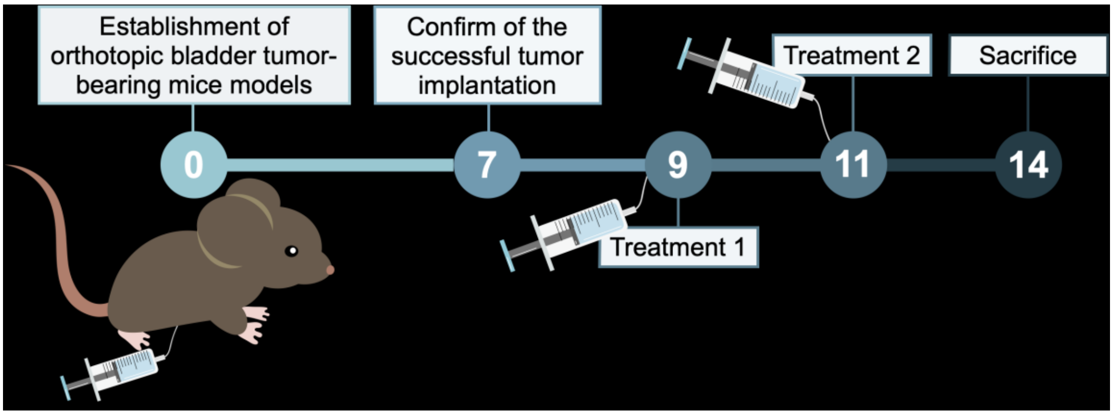

Figure S6. Schematic of experimental timeline for the animal study. The consequence of tumor implantation was examined on Day 7. Various treatments were performed on Day 9 and Day 11 for a total of two courses.

8. Expression of IP-10 (CXCL10) induced by B-MSN and MSN-SH(E)

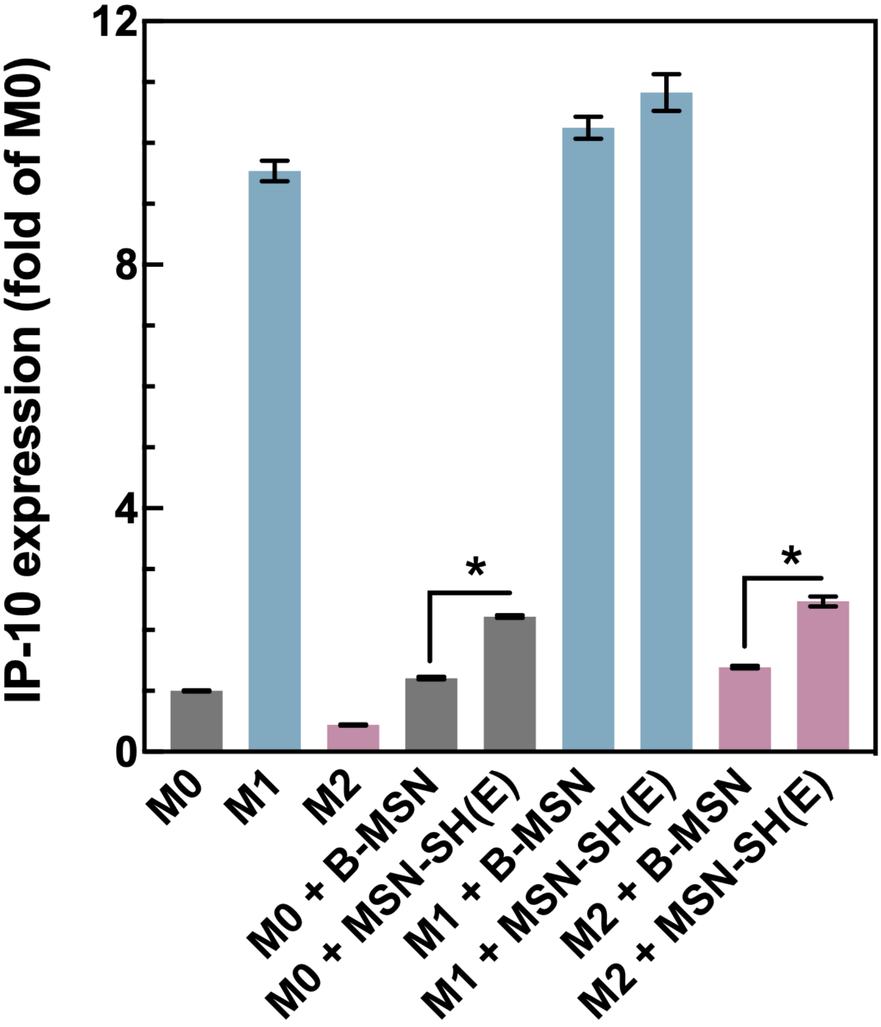

Figure S7. IP-10 (CXCL10) expression induced by B-MSN and MSN-SH(E). Pretreatment of IFN-γ + LPS and IL-4 was used for polarization of M1- and M2-like macrophages in the Transwell insert, respectively. The expression of IP-10 (CXCL10) from MBT-2 cells was determined by RT-qPCR. Macrophage pre-treated with only IFN-γ + LPS was used as positive control (M1). Cytokines were washed off before the addition of nanoparticles. All the results are shown as mean ± SD, n=3. (*$P < 0.05$).

Reference

[1] R. Benesch, R. E. Benesch, *PNAS* **1958**, *44* (9), 848.

[2] M. Lutolf, N. Tirelli, S. Cerritelli, L. Cavalli, J. Hubbell, *Bioconjugate Chemistry* **2001**, *12* (6), 1051.

[3] G. L.Ellman, *Archives of Biochemistry and Biophysics* **1959**, *82* (1), 70.

[4] J. W. Nelson, T. E. Creighton, *Biochemistry* **1994**, *33* (19), 5974.

[5] H. Schmidt, R. L. Krauth-Siegel, *Journal of Biological Chemistry* **2003**, *278* (47), 46329.

[6] N. Reckenfelderbäumer, R. L. Krauth-Siegel, *Journal of Biological Chemistry* **2002**, *277* (20), 17548.

[7] R. E. Hansen, D. Roth, J. R. Winther, *PNAS* **2009**, *106* (2), 422.

[8] A. Zeida, M. Trujillo, G. Ferrer-Sueta, A. Denicola, D. A. Estrin, R. Radi, *Chemical Reviews* **2019**, *119* (19), 10829.

[9] C. Leichner, M. Jelkmann, A. Bernkop-Schnuerch, *Advanced Drug Delivery Reviews* **2019**, *151*, 191.

[10] J. Sonnefeld, M. Lobbus, W. Vogelsberger, *Colloids and Surfaces A: Physicochemical and Engineering Aspects* **2001**, *195* (1-3), 215.

[11] D. Bermejo-Velasco, A. Azemar, O. P. Oommen, J. Hilborn, O. P. Varghese, *Biomacromolecules* **2019**, *20* (3), 1412.
